# Role of Apple Phytochemicals, Phloretin and Phloridzin, in Modulating Processes Related to Intestinal Inflammation

**DOI:** 10.3390/nu11051173

**Published:** 2019-05-25

**Authors:** Danuta Zielinska, José Moisés Laparra-Llopis, Henryk Zielinski, Dorota Szawara-Nowak, Juan Antonio Giménez-Bastida

**Affiliations:** 1 Department of Chemistry, University of Warmia and Mazury, 10-727 Olsztyn, Poland; dziel@uwm.edu.pl; 2 Group of Molecular Immunonutrition in Cancer, Madrid Institute for Advanced Studies in Food (IMDEA-Food), 28049 Madrid, Spain; moises.laparra@imdea.org; 3 Institute of Animal Reproduction and Food Research, Department of Chemistry and Biodynamics of Food, Polish Academy of Science, 10-748 Olsztyn, Poland; h.zielinski@pan.olsztyn.pl (H.Z.); d.szawara-nowak@pan.olsztyn.pl (D.S.-N.); 4 Group on Quality, Safety and Bioactivity of Plant Foods, Centro de Edafología y Biología Aplicada del Segura (CSIC), 30100 Murcia, Spain

**Keywords:** apple, phloretin, phloridzin, myofibroblasts, CCD-18Co, antioxidant activity, anti-inflammatory, antiglycation

## Abstract

Plant-derived food consumption has gained attention as potential intervention for the improvement of intestinal inflammatory diseases. Apple consumption has been shown to be effective at ameliorating intestinal inflammation symptoms. These beneficial effects have been related to (poly)phenols, including phloretin (Phlor) and its glycoside named phloridzin (Phldz). To deepen the modulatory effects of these molecules we studied: i) their influence on the synthesis of proinflammatory molecules (PGE_2_, IL-8, IL-6, MCP-1, and ICAM-1) in IL-1β-treated myofibroblasts of the colon CCD-18Co cell line, and ii) the inhibitory potential of the formation of advanced glycation end products (AGEs). The results showed that Phlor (10–50 μM) decreased the synthesis of PGE_2_ and IL-8 and the formation of AGEs by different mechanisms. It is concluded that Phlor and Phldz, compounds found exclusively in apples, are positively associated with potential beneficial effects of apple consumption.

## 1. Introduction

The intestine is permanently exposed to exogenous and endogenous environmental factors from dietary habits and metabolites from intestinal microbiota. In health conditions, the simultaneous interaction “microbiota–intestinal cells–food” is in a relatively stable physiological equilibrium. The latter can be disrupted due to the particular food composition characteristic of Western diets. Here, the presence of a large amounts of processed foods containing advanced glycation end products (AGEs) significantly contributes to imbalances in gut microbiota diversity (dysbiosis) and the development of intestinal inflammatory processes [1,2]. During chronic intestinal inflammation, intestinal cells undergo a constant activation increasing the expression of receptors (i.e., toll-like or AGEs receptors) [3,4]. These cellular response(s) and the production of chemokines allow the infiltration of immunocompetent cells, which in turn synthesize pro-inflammatory cytokines such as interleukin-1β (IL-1β). Notably, this uncontrolled environment promotes alterations and potential disruption of the intestinal barrier architecture and aggravates the inflammatory conditions [5].

An attractive approach to protect intestinal cells functionality and preserve adequate conditions of the intestinal barrier comes from the evidence reported into the benefits associated with an appropriate intake of plant-derived food [6]. Apples (*Malus* sp., Rosaceae) are one of the most widely cultivated fruits in the world. They are a significant part of human diet and have been ranked in the top five consumed fruits in the world. Evidence from in vivo animal studies suggests a protective role of apple consumption against intestinal inflammation partially modulated by (poly)phenolic compounds [7,8,9]. Apples have been identified as an important dietetic source of (poly)phenols [10], despite the variable concentration of these molecules between different apple varieties.

**Figure 1:**
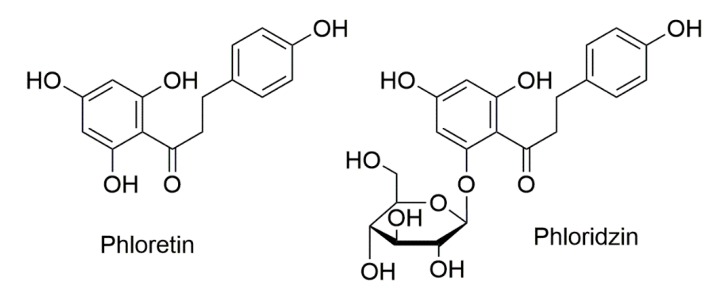
Chemical structures of phloretin and phloridzin (phloretin 2′-*O*-glucose).

Apple (polyphenols) can be classified into five major sub-classes where procyanindins represent the most abundant proportion (between 40% and 89%), followed by hydroxycinnamic acids, dihydrochalcones, flavonols, anthocyanins, and flavan-3-ols [11]. Phloridzin (Phldz) belongs to the chemical class of dihydrochalcones, phenylpropanoids with structures closely related to those of the immediate flavonoid precursors (chalcones). It consists of a C_6_–C_3_–C_6_ skeleton structure (two aromatic rings connected by a C3 chain) with a β-D-glucopyranose moiety attached at position 2’ (Figure 1). It has been reported that Phldz (phloretin 2′-*O*-glucose) is hydrolyzed by the intestinal lactase-Phldz hydrolase to form its aglycone, known as phloretin (Phlor). This undergoes phase-II metabolism generating conjugates (i.e., glucuronides) as the main derivatives, which are quantified in the mainstream plasmatic circulation in the range of μM concentration [12,13].

An important number of studies has investigated the anti-inflammatory activity of Phldz and Phlor to counteract lung [14,15] and/or liver [16] inflammation. Besides, at the intestinal level, the beneficial effects of Phlor and Phldz ameliorating intestinal inflammation [17,18] has been only inferential. The scarce number of studies are unable to provide a complete knowledge on the molecular mechanisms of action of Phlor and Phldz, which can help in designing appropiate clinical intervention trial to establish the nutritional recommendations for apple consumption. 

In view of the aforementioned, the main objective of this study was to evaluate some of the potential molecular mechanisms by which Phldz and Phlor may exert their anti-inflammatory effects at the intestinal level. Thus, we used the myofibroblasts of colon CCD-18Co as an in vitro model of intestinal inflammation to test whether Phldz and Phlor, at concentrations (50–10 μM) similar to those detected in vivo [12] are able to modulate the secretion of molecules (i.e., prostaglandins and chemokines) in response to IL-1β in myofibroblasts of colon. Also, we tested the capacity of both molecules to block the formation of the AGEs.

## 2. Materials and Methods

### 2.1. Materials

All chemicals were of analytical grade. Phlor and Phldz were supplied by Extrasynthese (Genay, France). 2,2-diphenyl-1-picrylhydrazyl (DPPH), 6-hydroxy-2,5,7,8-tetramethylchroman-2-carboxylic acid (Trolox), sodium azide, bovine serum albumin (BSA), D-glucose, methylglyoxal (MGO), 3-(2-pyridyl)-5,6-diphenyl-1,2,4-triazine-*p*,*p’*-disulfonic acid monosodium salt hydrate (ferrozine), IL-1β, Eagle’s minimum essential medium (EMEM), L-glutamine, nonessential amino acids, sodium pyruvate, sodium bicarbonate, penicillin/streptomycin, fetal bovine serum (FBS), Trypsin-EDTA (0.25–0.03%), and 3-(4,5-dimethylthiazol-2-yl)-2,5-diphenyltetrazolium (MTT) were purchased from Sigma (Sigma Chemical Co., St. Louis, MO, USA). DMSO was purchased from MERK Millipore (Darmstadt, Germany). Methanol, acetic acid (supra-gradient) and sodium acetate were from Merck KGaA, Darmstadt, Germany. Ultrapure water was purified with a Millipore Direct-Q UV 3 System (Merk Millipore, Bedford, MA, USA).

### 2.2. Apple Extracts’ Preparation for the Phldz and Phlor Analysis

The studied material consisted of 11 high-quality grade apple cultivars at their ripe period of growth. Early varieties of apples such as Antonówka, Delikates, Early Geneva, Papierówka, Paulared, Sunrise, Quinte as well as Gloster, Jonagored, Ligol and Rubinola cultivars, which are late apple varieties were harvested during the 2014 season. All fruits were purchased from the Experimental and Production Institute “Pozorty” Sp. z o.o. (Olsztyn, Poland). Fruit sample (10 apple fruits randomly selected) were peeled using a hand peeler (1–2 mm thickness), cored and cut into small pieces. The weighted apple flesh and peels were pooled separately, lyophilized, ground in a laboratory mill and stored at −20 °C. About 100 mg lyophilized flesh and peels of apple were extracted with 1 mL methanol (80% *v*/*v*) by 30 s sonication. Next, the mixture was vortexed for 30 s, again sonicated and vortexed, and centrifuged for 5 min (13,200 rpm). That step was repeated 5 times and the supernatant collected in 5 mL flask. Finally, all extracts were kept in dark-glass vials at −20 °C for analysis of Phlor and Phldz.

### 2.3. Analysis of Phlor and Phldz by HPLC-DAD-MS/MS

The analysis of Phlor and Phldz was performed using a micro-HPLC system LC200 Eksigent (Dublin, CA, USA). The micro-HPLC was connected in series to a mass spectrometer QTRAP 5500 (AB SCIEX, Toronto, Canada) equipped with a triple quadrupole, ion trap, and ion source of electrospray ionization (ESI). The analytical column was a Halo C18 column (50 mm × 0.5 mm, 2.7 µm i.d., Eksigent, USA). Eluent A was water/formic acid, 99.05/0.95 (*v*/*v*); eluent B was acetonitrile/formic acid, 99.05/0.95 (*v*/*v*). A gradient elution program was used as follows: 5–5–90–90–5–5 (% B) in 0–0.1–2–2.5–2.7–3 min. Before the chromatographic analysis apple extract was centrifuged (20 min, 13,000× *g*). A 2 µL aliquot of sample solution was injected, with flow rate of 15 µL/min, at a column temperature of 45 °C. Phlor and Phldz in the apple extracts were identified according to their MS/MS fragmentation spectrum (227.1/166.8/123.1 and 273) obtained in negative-ion mode. Optimal identification of Phlor and Phldz was achieved under the following conditions: curtain gas: 25 L/min, collision gas: 9 L/min, ionspray voltage: 5400 V (for positive-ion mode) and −4500 V (for negative-ion mode), temperature: 350 °C, 1 ion source gas: 35 L/min, 2 ion source gas: 30 L/min and entrance potential: 10 V (for positive-ion mode) and −10 V (for negative-ion mode). Phlor and Phldz were quantified from determined multiple reaction monitoring pairs (MRM) and calibration curves of external standards (the range of 10–1000 nM).

### 2.4. Cell Culture Conditions 

The myofibroblasts-like cell line CCD-18Co (American Type Culture Collection –ATTCC; Rockville, MD, USA) was maintained in EMEM (pH 7.2–7.4) supplemented with L-glutamine (2 mM), nonessential amino acids (0.1 mM), sodium pyruvate (1 mM), sodium bicarbonate (1.5 g/L), penicillin/streptomycin (100 U mL^−1^ and 100 mg mL^−1^, respectively) and FBS (10% *v*/*v*). Routinely, the cells were cultured at 6000 cells/cm^2^ in T75-flasks and incubated (37 °C, 5% CO_2_/95% air atmosphere, and constant humidity) for 4–5 days until reaching confluence ≥80%. The experiments were performed at population doubling levels (PDL) and passages ranging from 33 to 36 and 15 to 16, respectively.

### 2.5. Cell Viability Assay

An MTT assay allowed to determine whether Phlor and/or Phldz altered the cell viability of the myofibroblasts. Phlor and Phldz were diluted in DMSO to obtain stock solutions at 10 mM. The cells were treated with 50 μM Phlor or Phldz (0.5% DMSO, *v*/*v*), for 24 h, in the presence of 1 ng/mL IL-1β, and the cell viability was measured by determining the conversion of MTT to formazan. Cells were treated with an equivalent volume of DMSO (0.5%, *v*/*v*) as vehicle (Veh.) The experiment was repeated 3 times (*n* = 3) and each experiment was performed in triplicates.

### 2.6. Effect of Phlor and Phldz on the PGE_2_ Biosynthesis in IL-1β-Stimulated Myofibroblasts 

We initially determined whether Phlor and/or Phldz showed anti-inflammatory activity modulating the biosynthesis of PGE_2_ in IL-1β-stimualted myofibroblasts of colon. The compounds were diluted in DMSO as described above. Briefly, confluent cells (cultured in 96-well plates) were incubated in serum-deprived medium overnight followed by the treatment with 1 ng/mL IL-1

β in the presence or absence 50 μM Phlor and Phldz (0.5% DMSO, *v/v*) for 24 h. In parallel, control cells were treated with 0.5% DMSO (Veh.) in absence of IL-1β. The culture medium was removed and frozen at −80 °C until analysis. The samples diluted 100 times (1:100) were analyzed using an ELISA kit from Cayman (San Diego, CA, USA) to measure the PGE_2_ levels. In a second set of experiments, lower concentrations of Phlor (10 and 1 μM) were also assayed. The assays were repeated 3 times (*n* = 3) and each experiment was performed in triplicates.

### 2.7. Effect of Phlor on Cytokine Production in IL-1β-Stimulated Myofibroblasts

The levels of IL-8, IL-6 and MCP-1 (monocyte chemotactic protein-1) were also quantified in the culture medium obtained in the previous assay, while the expression of ICAM-1 was determined in the cell protein extract. The different cytokines were measured using enzyme-linked immunosorbent assay (ELISA) kits from Peprotech (Rocky Hill, NJ, USA). The assay was repeated 3 times (*n* = 3) and each experiment was performed in triplicates.

### 2.8. Inhibition of the Formation of the Advanced Glycation End Products (AGEs)

To determine the activity against the generation of AGEs, Phlor and Phldz were sequentially diluted in a small volume of DMSO and phosphate buffer (0.1 M, pH 7.4) to obtain 1 mM concentration of each compound (DMSO/phosphate buffer; 1:99; *v*/*v*). The inhibitory activity against formation of AGEs in the BSA/glucose and BSA/MGO systems was determined as previously described [19]. Aminoquanidine (AG), a hydrazine compound that prevents AGEs formation by trapping intermediates at the initial glycation stages [20], was used as positive control at 1 mM. Fluorescent intensity (BSA-glucose: Ex 330 nm/Em 410 nm and BSA-MGO: Ex 340 nm/Em 420 nm) was measured. Nine samples (*n* = 9) were run for each set and the inhibition of AGEs formation by Phlor and Phldz or AG solution was calculated as percentage of inhibition (fluorescence of the solution with Phlor or Phldz/fluorescence of the solution) × 100.

### 2.9. Antioxidant Activity

Phlor and Phldz were diluted in methanol (1 mM) and the concentration was confirmed by ultraviolet (UV) light measurement as previously described [21]. Based on a previous method reported [22], the antioxidant activity was measured using the DPPH radicals assay. DPPH radical scavenging activity (DPPH RSA), measured in a temperature-controlled spectrophotometer UV-160 1PC with CPS-Controller (Shimadzu, Japan), was expressed as mM of Trolox of nine independent experimens (*n* = 9).

### 2.10. Reducing Activity

A cyclic voltammetry (CV) assay was the method used to determine the reducing capacity described elsewhere [23]. Phldz and Phlor (diluted in MeOH as described above) were diluted (1:1 *v*/*v*) in Britton-Robinson (B-R) buffer (0.1 M, pH 6.0) to obtain a 250 µM solution. A G750 Gamry potentiostat (USA) was used to obtain the cyclic voltammograms of Phldz and Phlor. The potential range for the studied compounds was from −0.1 to 1.2 V, while for Trolox from −0.1 to 1.3 V at a scan rate of 0.1 V s^−1^. The results of nine independent experiments (*n* = 9) were expressed as mM of Trolox.

### 2.11. Ferric-Reducing/Antioxidant Power (FRAP) Activity

The ferric-reducing/antioxidant power (FRAP) assay was performed according to previous studies [24] The samples were diluted in MeOH as described above. A range of concentration from 0.034–0.612 mM of Trolox was used as standard curve. The results were expressed as mM of Trolox equivalent. The experiment was repeated up to 9 times (*n* = 9)

### 2.12. Chelating Activity

Ferrous ions chelating activity was measured by inhibition of the formation of Fe^2+^—Ferrozine complex after treatment of the tested compounds with Fe^2+^ [25]. A range of concentrations from 5 to 60 μM of Fe^2+^ ions was prepared as a standard curve. Ferrous chelation efficiency of Phldz and Phlor was expressed in % of 9 different replicates (*n* = 9).

### 2.13. Statistical Analysis

Results are given as the average ± standard deviation (SD) of *n* = 9 (for chemical analysis) or *n* = 3 (in vitro cellular assays) independent experiments. For normal distributed data, 1-way analysis of variance (ANOVA) followed by Dunnet´s post-hoc was used for data analysis. The significant differences were indicated as * *p* < 0.05, ** *p* < 0.01, and *** *p* < 0.001. The correlation was determined using the Pearson test.

## 3. Results

### 3.1. Phlor and Phldz Contents in Apple Peel and Flesh

The concentration of Phlor and Phldz quantified in the apple cultivars is shown in Table 1. The results revealed highly variable concentration ranges of Phldz (16.4–84.11 and 6.6–45.1 μg/g FW in peel and flesh, respectively) and Phlor (0.6–2.2 and 0.4–0.8 μg/g FW in peel and flesh, respectively). Other compounds such as catechin, epicatechin, chlorogenic acid, quercetin 3-glucoside, quercetin 3-arabinoside, quercetin 3-rhamnoside and cyanidin 3-galactoside were found at higher abundance than Phldz and Phlor. Only the levels of quercetin and rutin was similar to that of Phlor and Phldz (data not shown). Its concentration was about three-fold higher in peel than in flesh. The highest level of Phlor was quantified in the peel of Papierówka cultivar, except for Antonówka cultivar where similar concentrations were quantified both in peel and flesh (Table 1).

### 3.2. Inhibition of PGE_2_ Biosynthesis by Phlor and Phldz

**Figure 2:**
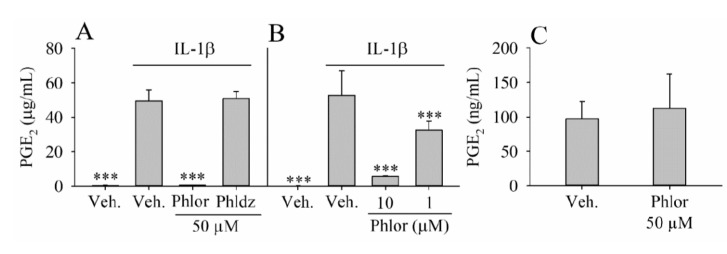
PGE_2_ level in culture medium of myofibroblast of colon CCD-18Co treated with (**A**) IL-1β (1 ng/mL) alone or in combination of Phlor or Phldz (50 μM); (**B**) IL-1β (1 ng/mL) alone or in combination of Phlor (10 and 1 μM); (**C**) Phlor (50 μM). The results are expressed as mean ± standard deviation (SD) obtained from 3 different experiments (*n* = 3). Statistically significant differences are showed as follows: *** *p* < 0.001.

The IL-1β-stimulated myofibroblats were treated with 50 μM Phlor and Phldz to analyze whether these compounds were able to modulate the synthesis of PGE_2_ (Figure 2). The treatment of cell cultures with IL-1β did not affect cell viability as values remained at 90% of controls (data not shown). Notably, cell cultures challenged by IL-1β dramatically increased the concentration of PGE_2_ (*p* < 0.001). The co-treatment with Phlor significantly (*p* < 0.001) ameliorated the effect of the IL-1β reducing the PGE_2_ levels to the control levels, whereas no effect was observed when the cells were co-treated with Phldz (Figure 2A). It was found a dose-effect relationship for Phlor; 10 μM decreased the PGE_2_ concentration about 90% (*p* < 0.001), whereas this reduction was 38% at 1 μM. Phlor (50 μM), in absence of IL-1β, did not have influence on the production of PGE2 by myofibroblasts (Figure 2C).

### 3.3. Effect of Phlor on Cytokines Biosynthesis in IL-1β-Stimulated Myofibroblasts

**Figure 3:**
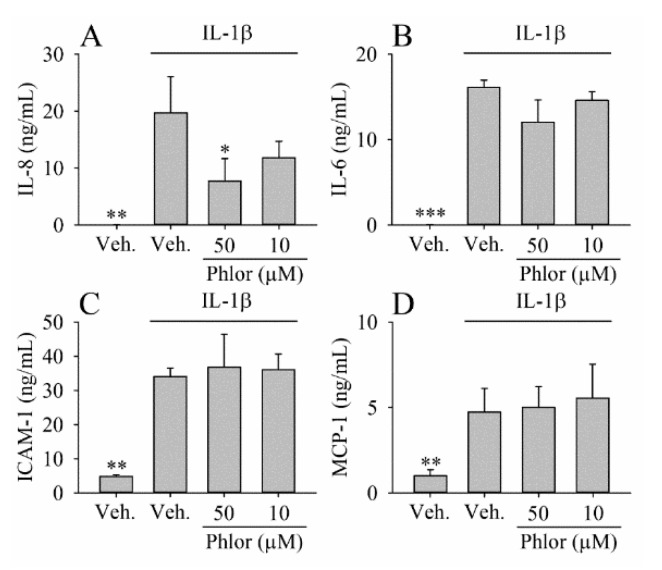
Level of cytokines and adhesion proteins analyzed in culture medium and protein extract, respectively, by enzyme-linked immunosorbent assay (ELISA). The myofibroblasts of colon were treated with IL-1β (1 ng/mL) alone or in combination with Phlor (50 and 10 μM) for 24 h and the level of IL-8 (**A**), IL-6 (**B**), ICAM-1 (**C**), and MCP-1 (**D**) determined. Statistically significant differences are shown as follows: * *p* < 0.05, ** *p* < 0.05, *** *p* < 0.001.

Based on the previous results, only those concentrations reducing the production of PGE_2_ more than 50% in relation to IL-1β-treated cells were studied. Phlor at 50 μM attenuated the concentration of IL-8 in the culture medium (by 61%; *p* < 0.05), whereas concentration at 10 μM of the reduction observed (by 40%) was not significant (Figure 3A). Besides, the up-regulation of IL-6 induced by IL-1β was slightly (*p* > 0.05) attenuated (about 25%) by Phlor at 50 μM, whereas concentration at 10 μM did not cause any effect (Figure 3B). Under both concentrations assayed, Phlor had no effect on the concentrations of ICAM-1 and MCP-1 (Figure 3C,D).

### 3.4. Inhibition of In Vitro AGEs’ Formation by Phlor and Phldz

**Figure 4:**
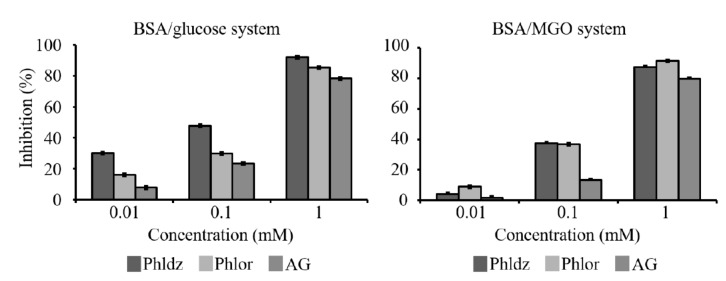
The concentration-dependent inhibitory effects of phloretin, phloridzin (phloretin 2′-*O*-glucose) and aminoguanidide (AG) against advanced glycation end products (AGEs) formation as measured in bovine serum albumin (BSA)/glucose and BSA/D-glucose, methylglyoxal (MGO) systems.

The inhibitory activity of Phlor and Phldz against formation of AGEs determined using the BSA/glucose and BSA/MGO assays is shown on Figure 4. The AG used (1 mM) as positive control exhibited an inhibitory capacity above 78% in the BSA/glucose and BSA/MGO models. Phlor and Phldz inhibited in a dose-dependent manner (from 0.01 to 1.0 mM) the formation of the AGEs, reaching similar inhibitory values or even higher than those of the positive control. These results clearly indicate that Phldz and Phlor display strong inhibitory capacity on the AGEs formation.

### 3.5. The Antioxidant, Reducing and Chelating Activity of Phlor and Phldz 

Phlor exhibited antioxidant and reducing activity between 3–5-fold higher than that of Phldz, but significantly lower of that for ascorbic and uric acids (Table 2). The DPPH RSA values for Phlor were 3-fold higher than those noted for Phldz (0.06 ± 0.01 mM Trolox), but 3- and 5-fold lower compared to ascorbic and uric acids. The FRAP values for Phlor and Phldz were 0.95 ± 0.02 and 0.18 ± 0.01, respectively. In contrast, Phldz showed higher chelating activity than Phlor, whose activity was 2-fold lower than ascorbic and uric acids. 

### 3.6. Reducing Power of Phlor and Phldz by Cyclic Voltammetry

**Figure 5:**
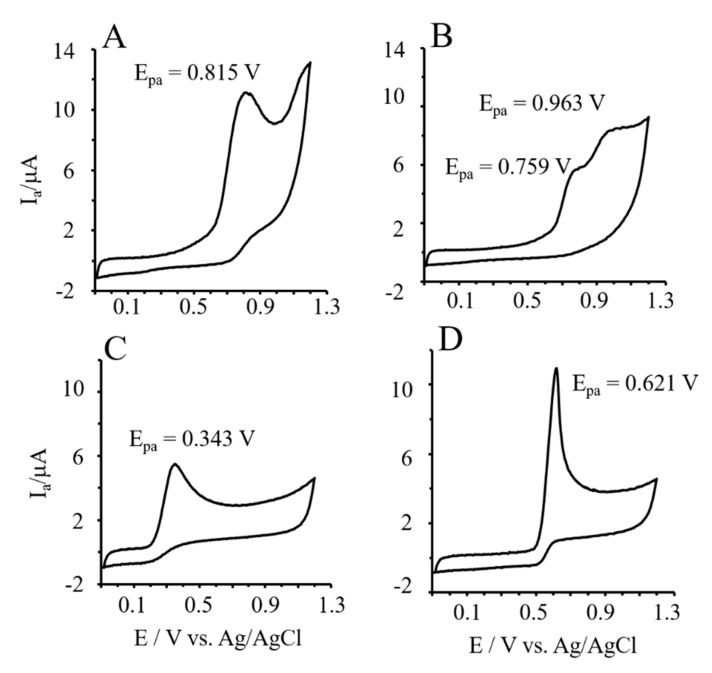
Cyclic voltammograms of 0.25 mM of standards solution (final concentration) of phloretin (**A**), phloridzin (**B**), L-ascorbic (**C**) and uric acid (**D**) in Britton–Robinson (B–R) buffer (0,1 M; pH 7,4) recorded from −100 to +1300 mV; scan rate 100 mV s^−1^.

The CV method based on the anodic peak potentials and the area under voltammograms was used for the characterization of the reducing activity of Phlor and Phldz (Figure 5). Standards solution (0.25 mM) of the studied compounds were prepared in 0.1 M Britton-Robinson buffer at pH 6.0, and cyclic voltammograms were recorded from −100 to +1200 mV; scan rate 100 mV s^−1^. The reducing power of Phlor was almost 2-fold higher than Phldz, but lower than that for ascorbic and uric acids (Table 2). The the anodic peak potentials (E_pa_) of the investigated compounds varied according to the following gradation: Phlor (0.815 V) > Phldz (0.759 V) > uric acid (0.621 V) > Trolox (0.346V) > L-ascorbic acid (0.343 V) (Figure 5). The higher E_pa_ values were associated with the lower reducing activity of the tested compound. The gradation of samples for the reducing activity provided by CV technique mirrored that obtained with the FRAP assay. Thus, both methods could be positively correlated (*r* = 0.88). In contrast, results obtained by CV displayed an inverse correlation with the chelating activity (*r* = −0.93).

## 4. Discussion

Food composition and the potential bioactivity of functional ingredients has received growing interest in order to allow the determination of adequate personalized nutritional recommendations to prevent or treat chronic diseases (i.e., intestinal inflammation). A significant number of studies approached the potential key role of polyphenols on the prognosis of intestinal inflammation. Here, a major proportion of the studies were focused on apple fruit and the polyphenol distribution (skin, pulp and seeds) in different apple cultivars as well as the major sub-classes of phenolic compounds in those [11,26,27,28,29]. Among these, dihydrochalcones (i.e., phloretin-2′-O-xyloglucoside and phloretin-2′-O-glucoside) have been exclusively described in apples. In particular, phloretin-2′-O-xyloglucoside and phloretin-2′-O-glucoside at concentrations of 63.7 and 35.5 μg/g FW, respectively, in raw apples, cultivar “Idared” [30]. Also, phloretin-2′-O-xyloglucoside and phloretin-2′-O-glucoside (Phldz) in skin and pulp of a rare apple from Marche region (Italy) [31]. Notably, the concentration of Phldz found in our study (16.4–84.11 and 6.6–45.1 μg/g FW in peel and flesh, respectively) was comparable to that previously reported [30,31]. Our results showed that Phldz and its aglycone Phlor are present in the peel and flesh of apples, whereas phloretin-2′-O-xyloglucoside was undetected, indicating that Phldz is the main dihydrochalcone present in the analyzed apples. These differences could be explained, at least in part, by differences in the methodologies used (i.e., methods of extraction or equipment). The exclusive presence of Phldz in apple fruit and its abundance are important factors to be considered (i.e., as an indicator for quality control and assurance) during the elaboration of different apple-based products. 

Relatively recent studies in rodents support the beneficial effects derived from apple consumption as a nutritional approach in the prevention [32] and treatment [33] of intestinal inflammation. The apple flavonoid Phlor has been proposed as one of the molecules involved in the anti-inflammatory effects, at the intestinal level, associated with apple consumption [17]. However, the molecular mechanisms have been poorly investigated. In vitro studies have associated the anti-inflammatory effect of Phlor to an increased level of the antioxidant enzyme glutathione in Caco-2 and HT-29 colon cancer cells [34], and downregulation of NF-κB and IL-8 in DLD1 colon cancer cells [35]. To deepen understanding of the mechanisms underlying the anti-inflammatory activity of Phlo and Phldz, in this study we used a cell model of human myofibroblasts of colon CCD-18Co, which are a widely approached non-cancerous colon cell model to investigate the anti-inflammatory effects of polyphenols.

Bioavailability studies in humans (ileostomy subjects) have reported that Phlor is a metabolite that can reach the intestine after apple juice or cider consumption [36,37]. However, these studies did not consider the effect of the colon microbiota due to the lack of colon of the subjects, and the concentrations documented are not representative of those achieved in vivo. In Wistar rats fed Phlor and Phldz enhanced diet (22 mg Phlor day^−1^; 88 mg Kg^−1^ body weight), the concentration of Phlor (aglycone and conjugates) in plasma reached concentrations up to 66.9 ± 19.4 μmol L^−1^ [12,38]. Based on these studies and following the recommendations given to design relevant in vitro assays [39] we decided to: i) use a representative cell model of human myofibroblasts, which can be in contact with dietary compounds under inflammatory conditions [40], and ii) use range of concentrations (1–50 μM) similar to those achieved in vivo (described above).

Prostaglandin E_2_ (PGE_2_) is a key molecule in the regulation of the inflammatory response at the intestinal level. High levels of PGE_2_ are related to the development of pathological events, including intestinal inflammation [41]. In our study, Phlor (but not Phldz) reduced dramatically the formation of PGE_2_ induced by IL-1β under experimental concentrations that can be achieved in vivo [12]. A possible mechanism to explain this effect could be a receptor-mediated anti-inflammatory effect. Polyphenols such as resveratrol have shown ability to act as antagonist/partial agonist on estrogen receptors [42]. The incubation of the cells with 50 μM Phlor, in absence of IL1β, showed no changes in the PGE_2_ levels, indicating a lack of partial agonist action. However, based on these results, the competitive binding Phlor/IL1β receptor cannot be ruled out. A possible further mechanism can be found in previous studies describing the fact that phenolic compounds (such as urolithins) reduce the biosynthesis of PGE_2_ interfering with the activation of NF-κB/MAPK activation and downregulating the COX-2 expression [43]. We will take these findings in future studies as a reference to test whether Phlor exerts its attenuating effects on PGE_2_ formation via inhibition of NF-κB/MAPK activation and COX-2 expression. 

Besides PGE_2_, additional key molecules involved in the intestinal inflammatory response are IL-6, ICAM-1, MCP-1, and IL-8. These molecules are responsible of the regulation of the chemoattraction, infiltration and adhesion of immune cells to the inflammatory site [44]. Similar to the studies describing a reduction of the IL-8 levels in lung cancer cells co-treated with IL-1β and Phlor (30–100 μM) [45], we showed a significant decrease of IL-8 level at 50 μM Phlor, and a non-significant tendency to reduce its biosynthesis at 10 μM. The stimulating effect of PGE_2_ on IL-8 biosynthesis in fibroblasts [46,47] provides evidence indicating that the effect of Phlor on IL-8 is mediated via regulation of the PGE_2_ levels. Thus, the effect of Phlor on the PGE_2_ and IL-8 suggests that Phlor might be one of the molecules responsible (together with other apple´s polyphenols) for the benefits reported after apple (or apple-derived products) consumption. However, Phlor did not affect the levels of IL-6, ICAM-1, and MCP-1 at the concentrations investigated. In contrast, Phlor has been shown effective to reduce the levels of IL-6 in LPS-treated macrophages [48], ICAM-1 in IL-1β-treated epithelial lung cells [49] and MCP-1 in colon and immune cells [35]. These results indicate that the effects of the apple polyphenols, including Phlor vary depending on numerous factors such as the cell line investigated, the specific pro-inflammatory cytokine tested, as well as the experimental conditions.

AGEs have been placed in the spotlight as dietary factors that can be accumulated in the gastrointestinal tract stimulating the inflammatory response [2]. In the past few years, a series of studies have showed a reduced susceptibility to the intestinal inflammation development via blockade of the AGEs’ interaction with their receptor (RAGE) [4,50]. In vivo animal studies have associated the consumption of Phlor with a reduction in the AGEs formation [51]. Hitherto, the mechanism(s) underlying this effect remains elusive. The possible mechanisms underlying the role of Phlor and Phldz in AGEs-mediated intestinal inflammation are diverse but mainly refer to their competence with lysine and arginine [52], less capacity to form MGO adducts at positions 3’ and 5’ of the A-ring [52,53], and antioxidant activity [54,55]. Previous in vitro studies focused on the MGO as the main precursor of AGEs [54,55], overlooking other possible important sources such as glucose. Thus, in this study we used two different in vitro models: MGO/BSA and glucose/BSA models. While the MGO/BSA model showed similar inhibitory activity against the AGEs formation, the glucose/BSA model displayed a higher inhibitory effect of Phldz than Phlor. These results highlight the importance of using different in vitro models to more accurately determine the capacity of different compounds to inhibit AGEs formation.

The apple’s polyphenols may act as reducing agents, hydrogen donors, free radical scavengers and singlet oxygen quenchers, as well as exhibiting antioxidant activity via chelation of metal ions [56]. The relevant antioxidant potential elicited by Phlor and Phldz [57] offers an additional mechanism impeding the formation of AGEs, thus ameliorating the intestinal inflammation complications. Human studies have described an increase of the antioxidant activity in human serum after apple consumption (300 mL apple juice—comparable to 5 apples—single dose) [58], what could be partially associated with the levels of Phlor and its derivatives detected in plasma [38]. However, in a different human study, the high antioxidant capacity of different apple extracts and their polyphenols did not parallel equivalent ex vivo antioxidant effects in plasma of volunteers after the consumption of apples (5 apples, single dose) [59]. The use of different methods to estimate the antioxidant activity of polyphenols (rather than a single-based approach) could improve our understanding of the antioxidant activity of Phlor and Phldz. Thus, in this study we combined traditional methods (FRAP or DPPH) with more recent techniques, such as cyclic voltammetry (not used previously to test the antioxidant activity of Phlor and Phldz). Our results showed a higher antioxidant and reducing activity in Phlor compared to its glycoside Phldz (probably due to the glucose molecule bound to the 2’ OH-group). These results may indicate a higher grade of contribution of Phlor to hinder AGE generation through antioxidant activity than its glycoside.

## 5. Conclusions

Overall, the results of this study contribute to expand knowledge in relation to the role of Phlor and Phldz as molecules responsible for potential benefits on intestinal inflammation that are associated with apple consumption. The mechanisms described provide evidence that these compounds mainly exhibit antioxidant effects and to a much lesser extent influence cytokine-driven inflammation. The effectivity of Phldz and Phlor from apples improving the quality of anti-inflammatory response(s) at the intestinal level can contribute to efforts to tailor nutritional interventions for large groups of populations. However, because of the relevance for health and social impact of chronic diseases derived from the inflammation-drive impairment of intestinal function, additional in vitro, animal and human studies research is needed, which will help establish the basis for the elaboration of new and more effective functional foods (i.e., apple-enriched products).

## Figures and Tables

**Table 1:** Concentration of phloretin and phloridzin identified by the HPLC-DAD-MS/MS analysis in the peel and flesh of different apple cultivars.

Apple Cultivar/Compound	Phloretin		Phloridzin	
	Peel	Flesh	Peel	Flesh
Quinte	0.96 ± 0.02 ^d^	0.72 ± 0.03 ^b^	23.91 ± 0.48 ^d^	18.89 ± 0.98 ^d^
Jonagored	0.91 ± 0.08 ^d^	0.42 ± 0.01 ^g^	24.65 ± 1.63 ^d^	7.17 ± 0.17 ^h^
Early Geneva	0.85 ± 0.02 ^e^	0.72 ± 0.01 ^b^	24.45 ± 0.49 ^d^	20.63 ± 0.80 ^c^
Paulared	0.64 ± 0.01 ^g^	0.51 ± 0.02 ^e^	16.43 ± 0.76 ^e^	10.37 ± 0.36 ^f^
Sunrise	1.38 ± 0.08 ^b^	0.60 ± 0.03 ^d^	48.08 ± 0.80 ^b^	14.68 ± 0.84 ^e^
Gloster	1.32 ± 0.02 ^b^	0.41 ± 0.00 ^g^	43.45 ± 2.59 ^c^	5.86 ± 0.16 ^i^
Delikates	0.73 ± 0.03 ^f^	0.47 ± 0.01 ^ef^	18.30 ± 1.74 ^e^	9.14 ± 0.06 ^g^
Papierówka	2.25 ± 0.05 ^a^	0.75 ± 0.03 ^b^	84.10 ± 1.60 ^a^	23.46 ± 0.77 ^b^
Rubinola	1.24 ± 0.05 ^c^	0.65 ± 0.03 ^c^	42.90 ± 3.21 ^c^	18.09 ± 0.22 ^d^
Antonówka	1.20 ± 0.01 ^c^	1.38 ± 0.08 ^a^	23.37 ± 0.45 ^d^	45.05 ± 1.79 ^a^
Ligol	0.66 ± 0.04 ^g^	0.43 ± 0.01 ^fg^	16.51 ± 0.73 ^e^	6.61 ± 0.14 ^hi^

Each value represents mean ± standard deviation (*n* = 3). Different letters in the same column indicate significant differences by the analysis of variance (ANOVA) test (*p* < 0.05). Results are expressed in μg/g FW.

**Table 2:** The antioxidant, reducing and chelating activity of phloretin and phloridzin in comparison to L-ascorbic and uric acids.

Compound/Assay	Antioxidant Activity (mM Trolox)	Reducing Activity (mM Trolox)		Chelating Activity (%)
	DPPH RSA	FRAP	CV	FZ
Phloretin	0.19 ± 0.01 ^c^	0.95 ± 0.02 ^c^	0.56 ± 0.02 ^b^	3.78 ± 0.47 ^b^
Phloridzin	0.06 ± 0.01 ^d^	0.18 ± 0.01 ^d^	0.36 ± 0.03 ^c^	3.86 ± 0.34 ^a^
L-ascorbic acid	0.70 ± 0.01 ^b^	1.13 ± 0.02 ^b^	1.04 ± 0.01 ^a^	1.79 ± 0.36 ^c^
Uric acid	1.08 ± 0.01 ^a^	1.27 ± 0.01 ^a^	0.98 ± 0.01 ^a^	0.99 ± 0.11 ^d^

Results were provided by DPPH RSA—DPPH radical scavenging activity assay; FRAP—ferric-reducing/antioxidant power assay; CV—cyclic voltammetry assay; FZ—ferrozine assay. Data are expressed as means ± standard deviation (*n* = 9). Means in a column related to a respective assay followed by the different letters are significantly different (*p* < 0.05).
